# Evaluating Native *Bacillus* Strains as Potential Biocontrol Agents against Tea Anthracnose Caused by *Colletotrichum fructicola*

**DOI:** 10.3390/plants13202889

**Published:** 2024-10-15

**Authors:** Meixia Chen, Hui Lin, Weifan Zu, Lulu Wang, Wenbo Dai, Yulin Xiao, Ye Zou, Chengkang Zhang, Wei Liu, Xiaoping Niu

**Affiliations:** 1 College of Biological Science and Engineering, Industry and University Research Cooperation Demonstration Base in Fujian Province, Ningde Normal University, Ningde 352100, China; 52305043059@fafu.edu.cn (H.L.); wenbodaisy@163.com (W.D.); xyl_1110@163.com (Y.X.); 15259166927@163.com (Y.Z.); shin_ichi@126.com (C.Z.); liuwei0591@126.com (W.L.); 2 College of Life Science, Fujian Agriculture and Forestry University, Fuzhou 350002, China; 5220543036@fafu.edu.cn (W.Z.); luluwanghn@163.com (L.W.)

**Keywords:** disease resistance, microbial agents, tea plant, 16S rRNA, fengycin

## Abstract

Anthracnose of the tea plant (*Camellia sinensis*), caused by *Colletotrichum* spp., poses a significant threat to both the yield and quality of tea production. To address this challenge, researchers have looked to the application of endophytic bacteria as a natural alternative to the use chemical pesticides, offering potential for enhancing disease resistance and abiotic stress tolerance in tea plants. This study focused on identifying effective microbial agents to combat tea anthracnose caused by *Colletotrichum fructicola*. A total of 38 *Bacillus*-like strains were isolated from the tea rhizosphere, with 8 isolates showing substantial inhibitory effects against the mycelial growth of *C. fructicola*, achieving an average inhibition rate of 60.68%. Among these, strain T3 was particularly effective, with a 69.86% inhibition rate. Through morphological, physiological, and biochemical characterization, along with 16S rRNA gene phylogenetics analysis, these strains were identified as *B. inaquosorum* (T1 and T2), *B. tequilensis* (T3, T5, T7, T8, and T19), and *B. spizizenii* (T6). Biological and molecular assays confirmed that these strains could induce the expression of genes associated with antimicrobial compounds like iturin, fengycin, subtilosin, and alkaline protease, which effectively reduced the disease index of tea anthracnose and enhanced tea plant growth. In conclusion, this study demonstrates that *B. inaquosorum*, *B. tequilensis*, and *B. spizizenii* strains are promising biocontrol agents for managing tea anthracnose.

## 1. Introduction

Tea (*Camellia sinensis* L.) is a highly valuable woody perennial crop, crucial to the economies of tropical and subtropical regions such as China, India, Kenya, and Sri Lanka [1,2]. The tender shoots of tea plants, rich in metabolites beneficial to human health, are specifically cultivated for tea production [1,3]. Unfortunately, tea anthracnose, caused by *Colletotrichum* spp., is prevalent in the major tea-growing regions of China, significantly impacting tea yield and quality [1,3,4]. The disease first presents as small, water-soaked lesions on young leaves and twigs, progressing to larger, dark-brown, necrotic lesions [1,5,6]. Severe infections can lead to defoliation of both young and mature leaves, causing a substantial 30–50% reduction in tea yield [1,3].

Currently, the methods of managing tea anthracnose disease consist of prevention and treatment [7]. One effective strategy for controlling this disease is the breeding and cultivation of disease-resistant plants [3]. Previous reports have highlighted the resilience of the Zhongcha 108 cultivar against *Colletotrichum camelliae*, showing both disease tolerance and the production of high-quality green tea [8]. However, tea plant breeding is challenging due to its long growth cycle and limited breeding efficacy [8,9]. To combat pathogens and manage tea anthracnose, tea growers have often resorted to using various synthetic chemical fungicides, which has led to the development of fungicide-resistant pathogens and raised concerns over their adverse impacts on the environment and human health [10]. In addition, excessive usage of agrochemicals has led to serious negative impacts, such as soil pollution and degradation, water contamination, accumulation of chemical residues in the food chain, and harm to non-target organisms [10]. To address these challenges, a proactive solution involves the development of resistant tea cultivars that not only enhance tea plant yield and quality but also promote environmentally friendly biocontrol strategies as viable alternatives to chemical interventions [1,10]. Another promising approach is the utilization of biological control agents that can combat plant pathogens through mechanisms such as antibiosis, mycoparasitism, nutrient competition, and induction of resistance. Embracing biological control methods aligns more closely with current agricultural sustainability goals [11]. Therefore, the research focus on developing efficient and safe biological agents for the prevention and management of tea anthracnose disease is of paramount importance.

Presently, various strains of fungi, bacteria, and actinomycetes have been harnessed as biocontrol agents for combatting tea anthracnose disease [3,12,13]. Of these, bacteria, in particular, have shown promising biocontrol efficacy and commercial potential due to their ability to be easily cultured in fermentation culture, to colonize both the plant rhizosphere and endosphere, and to exhibit high diversity [14]. Among the bacterial species, *Bacillus* spp. stand out as rhizosphere-dwelling, growth-promoting bacteria with biocontrol characteristics. They are known for being non-pathogenic to plants, adept at colonizing diverse host plants, displaying high biocontrol effectiveness, and causing minimal environmental impact [15]. *Bacillus* spp. has been found to secrete plant growth-promoting substances like the hormone IAA, siderophores, and extracellular polysaccharides, while also possessing the abilities to fix nitrogen, solubilize phosphorus, and produce various antimicrobial compounds such as lipopeptides and hydrolytic enzymes [16,17,18,19,20].

Despite their beneficial characteristics, the investigation of endophytic bacteria isolated from tea plants for biocontrol applications has been limited. This study reports the isolation of endophytic *Bacillus* spp. from the rhizosphere soil of tea plants and the evaluation of their effectiveness against *C. fructicola*. Through morphological, physiological, and biochemical analyses, alongside the sequencing of 16S rRNA genes, the strains were identified as *B. inaquosorum*, *B. tequilensis,* and *B. spizizenii*. Further experiments, including plate confrontation assays and PCR cloning, confirmed the biocontrol properties of these isolates, their growth-promoting activities, and the presence of antimicrobial genes.

## 2. Results

### 2.1. Screening of Antagonistic Strains

**Figure 1:**
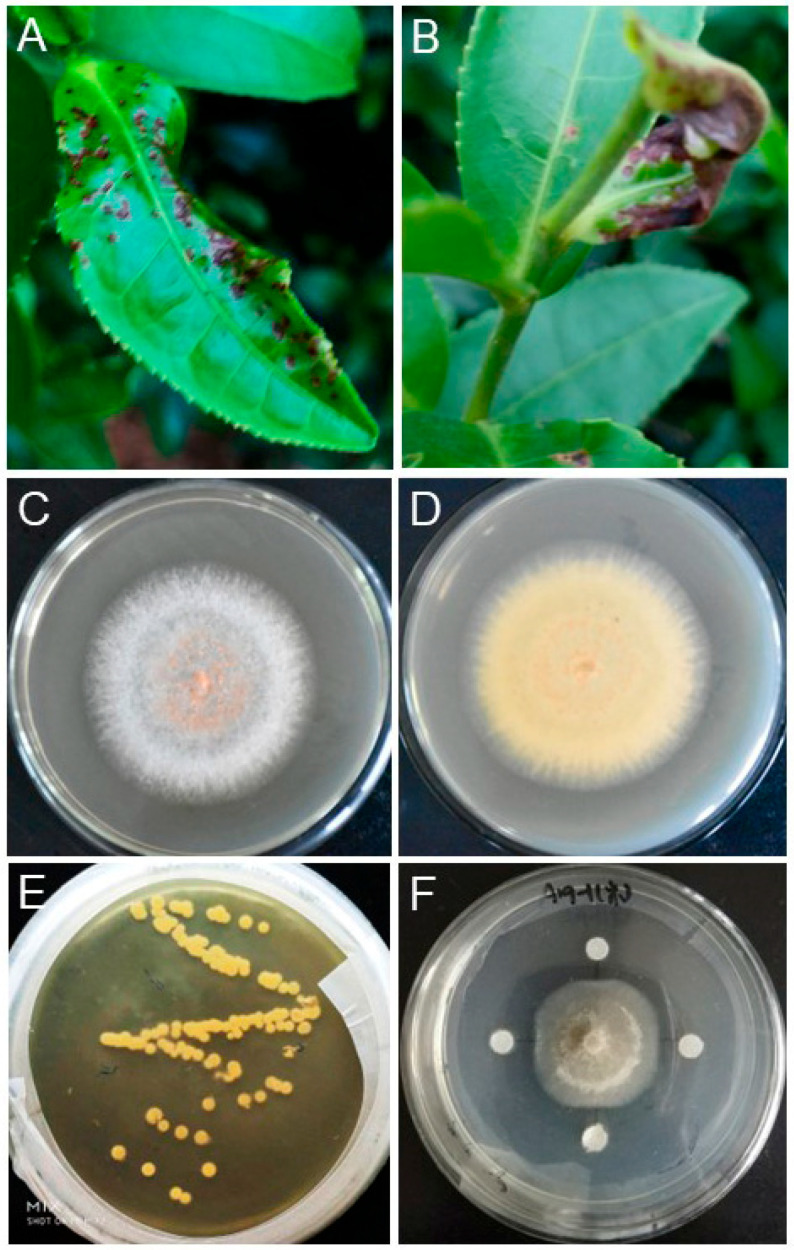
Screening of *bacillus* isolates against tea anthracnose pathogen *C. fructicola* strain N. (**A**,**B**) Symptoms of anthracnose disease on leaves of tea plant in the field. (**C**,**D**) Upper and reverse view of tea anthracnose pathogen *C. fructicola* strain N on PDA plate at 37 °C for three days. (**E**,**F**) Screening of *Bacillus* isolates from the rhizosphere soil of tea plants. Morphological characteristics of isolates (**E**) and their antagonistic effect against tea anthracnose pathogen *C. fructicola* on PDA plates at 37 °C for two days (**F**).

A total of 38 bacterial isolates were obtained from the rhizosphere soil of tea plants and labeled as T1 to T38. Initially, these isolates were classified as *Bacillus* based on their colony characteristics (Figure 1E). Subsequently, they were screened in vitro for antagonistic activity against the target pathogen *C. fructicola* strain N [21]. In confrontation cultures, 18 isolates demonstrated antibacterial properties by inhibiting the growth of *C. fructicola* strain N. Notably, eight isolates exhibited significant inhibition against pathogens, forming distinct inhibition zones (with a diameter of 3 mm) in LB agar plates (Figure 1F, Table 1). Among these, *Bacillus* strain T1 displayed the highest inhibition, followed by strains T2, T3, T4, T5, T7, T8, and T19. Consequently, eight isolates (T1, T2, T3, T4, T5, T7, T8, and T19) were chosen for their effectiveness in inhibiting the pathogen.

### 2.2. Identification of Bacillus-like Isolates

**Figure 2:**
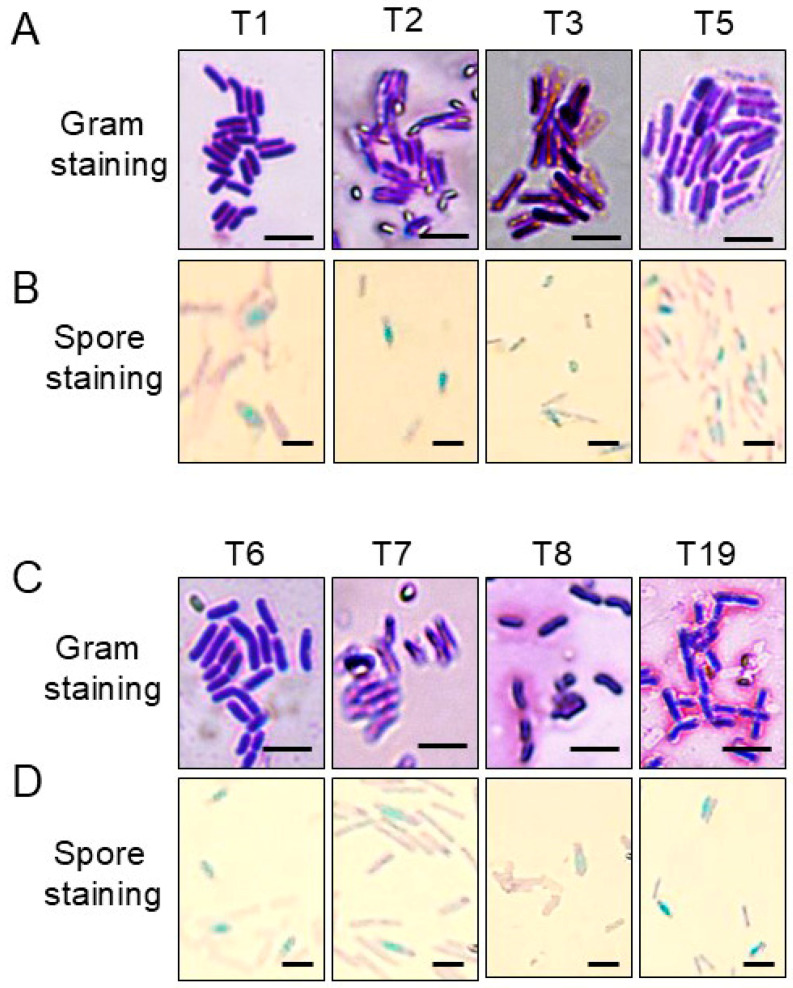
Morphological characteristics of the *Bacillus* isolates. Gram staining (**A**,**C**) and spore staining (**B**,**D**) of eight biocontrol strains. Size bars = 10 μm.

As depicted in Figure 2, the Bacillus-like strains cultured on PDA plates exhibited similar characteristics, forming mucoid colonies with a yellow hue. Microscopic examination revealed that these bacterial isolates were Gram-positive, rod-shaped, and capable of producing ellipsoidal spores with a bulging sporangium (Figure 2A,B). Moreover, results from physiological and biochemical assays indicated that these isolates tested positive for glycohydrolase (glucosidase, galactosidase, and β-xylosidase, with exception of strain T19), amino acid hydrolase (alanine arylamidase, glycine arylaminase, leucine arylamidase, phenylalanine arylamidase, and tyrosine arylamidase, with exception of strain T19), and esculin hydrolyse (Table 2). These strains, except for strain T19, also exhibited acid production when using D-glucose, D-mannose, D-mannitol, inositol, inulin, and pyruvate as substrates (Table 2). With exception of strain T19, they also demonstrated the ability to utilize polymyxin and grow in 6.5% NaCl, and strains T1, T2, and T3 showed resistance to kanamycin but showed no metabolic activity with putrescine for all eight isolates. Except for strain T2 and T7, these strains also showed no tolerance to oleandomycin phosphate (Table 2). Interestingly, strain T19 exhibited the activities of pyrrolidonyl arylamidase and A-P-P arylamidase, strains T1, T2, and T7 produced alanine arylamidase, and strain T3 yielded aspartate arylamidase (Table 2).

**Figure 3:**
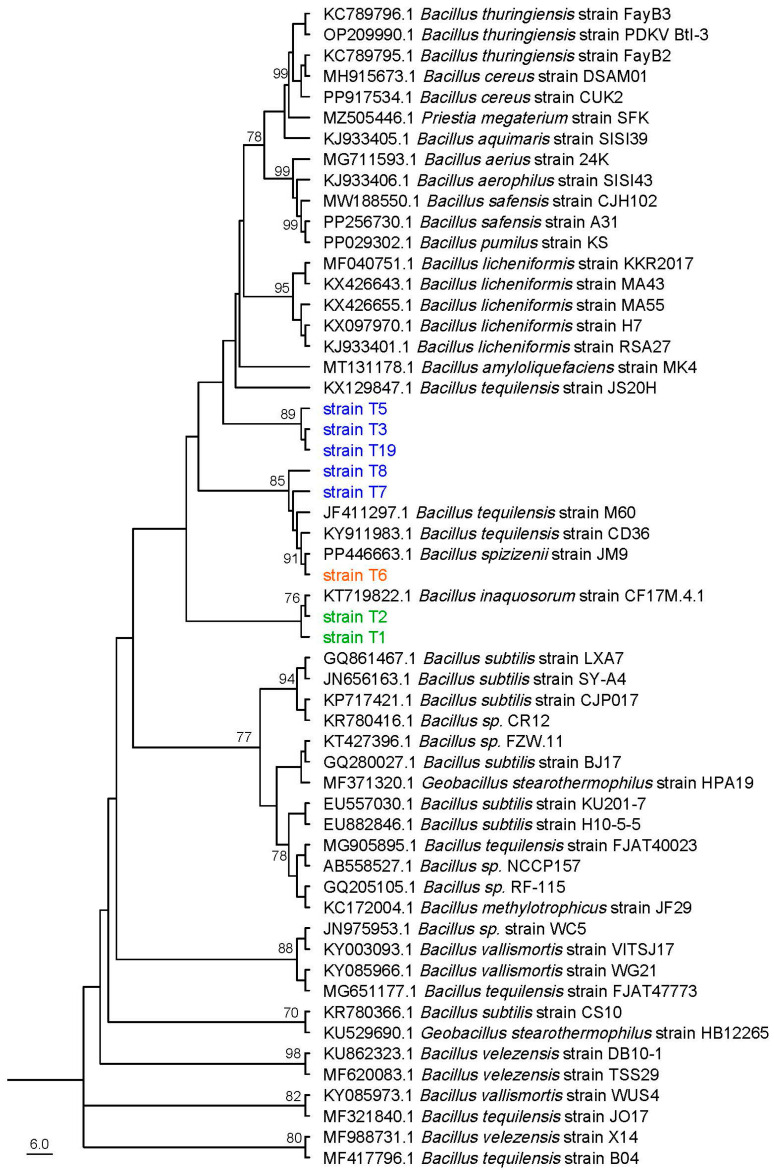
Maximum likelihood phylogenetic tree for eight strains and *Bacillus* spp. inferred from 16S rRNA gene sequences using MEGA 11. The numbers at the nodes indicate the bootstrap support calculated from 1000 replications, with values ≥ 70% displayed.

To determine their molecular identities, these isolates were analyzed using the housekeeping gene 16S rRNA gene sequences. Phylogenetic tree analysis conducted with MEGA v.11 software, based on alignments of 16S rRNA sequences, revealed that isolates T1 and T2 clustered with *B. inaquosorum*, with a bootstrap value of 76, while strains T3, T5, T7, T8, and T19 clustered with *B. tequilensis*, with bootstrap value of 89, and strain T6 was found to cluster with *B. spizizenii*, with bootstrap value of 91 (Figure 3). Additonally, a BLAST analysis of the 16S rRNA gene sequences from isolations T1 and T2 showed 100.0% and 98.59% homology to that of *B. inaquosorum* (KT719822), respectively. The 16S rRNA gene sequences of strains T3, T5, T7, T8, and T19 exhibited 99.22, 99.23, 98.27, 98.33, and 99.23% homology to *B. tequilensis*, respectively, while strain T6 showed 99.23% homology to *B. spizizenii*. Based on the combination of physiological characteristics and molecular identification, the bacterial isolates were successfully classified and characterized as Bacillus subspecies.

### 2.3. Evaluation of Antifungal Effect of Bacillus Strains

**Figure 4:**
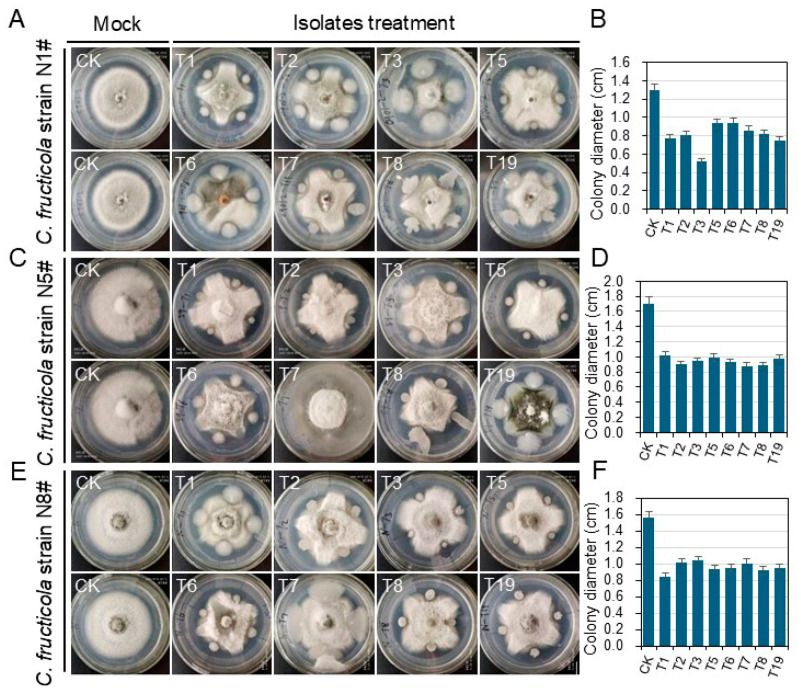
Inhibitory activity of *Bacillus* isolates against the *C. fructicola* strains. The inhibitory effects of eight *Bacillus* isolates on *C. fructicola* strain N1^#^ (**A**,**B**), *C. fructicola* strain N5^#^ (**C**,**D**), and *C. fructicola* strain N8^#^ (**E**,**F**). The photograph on the left of each panel represents the mock control (CK), and the images on the right display the inhibitory effects of *Bacillus* isolate treatment.

To evaluate the inhibitory effect of Bacillus strains on tea anthracnose disease, in vitro experiments were carried out by co-culturing *C. fructicola* with eight different Bacillus isolates. As depicted in Figure 4, the Bacillus strains displayed a high level of antagonistic activity against *C. fructicola* strains. Among these isolates, strain T3 exhibited the highest degree of antagonistic activity, followed by T19, T1, and T2, against the tea anthracnose pathogen *C. fructicola* strain N1^#^ (Figure 4A,B). Strain T7 displayed the greatest level of antagonistic activity against the tea anthracnose pathogen *C. fructicola* strain N5^#^ (Figure 4C,D). Strains T1, T5, and T6 demonstrated considerable levels of antagonistic activity against the tea anthracnose pathogen *C. fructicola* strain N8^#^ (Figure 4E,F). Additionally, the average inhibitory rates of the eight Bacillus strains against the tea anthracnose pathogen *C. fructicola* were above 60% (Table 3).

**Figure 5:**
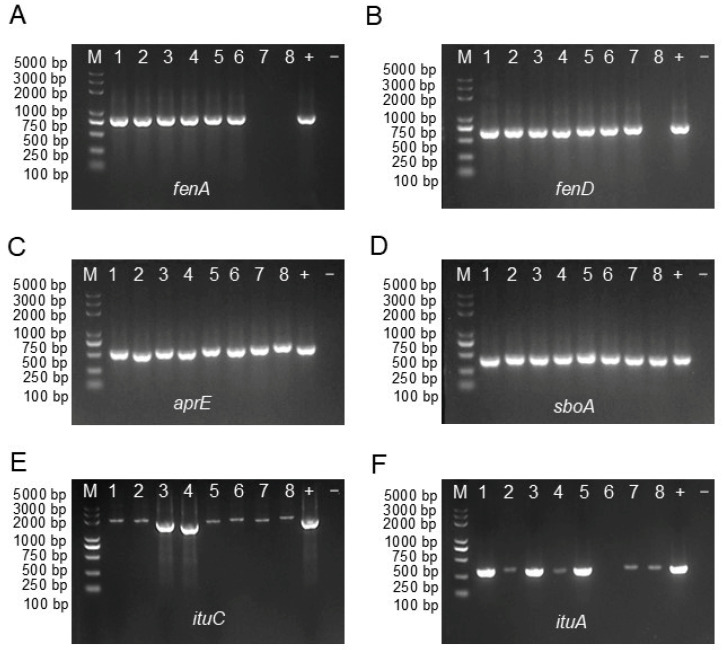
The PCR amplification of genes involved in the production of antimicrobial compounds using genomic DNA of 8 *Bacillus* isolates was verified by a 1% agarose gel. Bands corresponding to the *fenA* (**A**) and *fenD* (**B**) genes were associated with fengycin, *aprE* (**C**) with alkaline protease, *sboA* (**D**) with subtilosin, and *ituC* (**E**) and *ituA* (**F**) with iturin. M denotes the size marker, while numbers 1–8 correspond to strains T1, T2, T3, T5, T6, T8, and T19, respectively. + represents positive control, using DNA from *Bacillus subtilis* as a template, and − represents negative control, using ddH_2_O as a template.

To investigate the antimicrobial activities of these strains, genes associated with antimicrobial activities, such as aprE, ituA, ituC, fenA, fenD, and sboA, were amplified using specific primers, and the presence of these genes was verified by 1% agarose gel electrophoresis of the PCR products. The results revealed that genes responsible for biosynthesis of the alkaline protease (aprE) and subtilosin-A (sboA) were detected in these eight strains (Figure 5C,D). Genes encoding iturin antimicrobial compounds (ituA and ituC) were identified in almost all strains (Figure 5E), except for strain T6 for ituA (Figure 5F). While genes for the biosynthesis of fengycin (fenA) could not be identified in strains T8 and T19 (Figure 5A), fengycin (fenD) was found in strain T19 (Figure 5B). These results indicated that Bacillus strains exhibited diverse antibacterial properties, including glucanase secretion and protease activity, as well as the synthesis of antimicrobial substances such as iturin, fengycin, and nonribosomal polypeptide synthase.

### 2.4. In Vivo Efficacy against Tea Anthracnose

**Figure 6:**
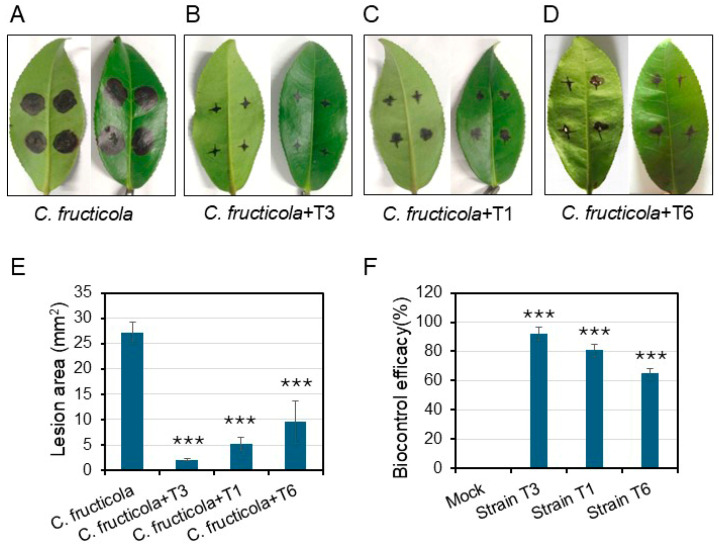
Biocontrol effect of *Bacillus* strain *B. inaquosorum* T1, *B. tequilensis* T3, and *B. spizizenii* T6 treatments on tea anthracnose on the tea leaves caused by *C. fructicola.* (**A**–**D**) Disease incidence of four leaf groups inoculated with *C. fructicola* and treated with different *Bacillus* strains. (**E**) Disease lesion areas and (**F**) control efficacy were calculated after 10 days of inoculation with the pathogen. *** indicates significant differences between treatments, according to one-way ANOVA (*p* < 0.05).

In a greenhouse pot experiment, the biocontrol efficacy of *Bacillus* strains *B. inaquosorum* T1, *B. tequilensis* T3, and *B. spizizenii* T6 was assessed against the pathogenic strain *C. fructicola* N on tea leaves. Symptoms, including disease incidence, lesion size, and control efficacy, were evaluated on the 10th day after inoculation. The control plants, inoculated only with *C. fructicola* strain N, exhibited a lesion area of 27.2 mm^2^, resulting in considerable plant mortality (Figure 6A–C). In contrast, plants treated with the bacteria displayed significantly reduced symptom severity. Notably, *B. tequilensis* strain T3 exhibited a lesion area of 2.1 mm^2^, followed by *B. inaquosorum* T1, at 5.2 mm^2^, and *B. spizizenii* T6, at 9.6 mm^2^, corresponding to biocontrol efficacies of 92.3%, 80.8%, and 64.8%, respectively (Figure 6A–C).

## 3. Discussion

Biological control stands out as a highly effective and eco-friendly approach for managing soil-borne fungal diseases [14,22]. Endophytic bacteria, which naturally inhabit most plants, have the ability to permanently and robustly reside in their host plants [22,23]. These endophytic bacteria serve as an excellent source for developing effective biocontrol agents [11,24]. Moreover, their ability to be transmitted through seeds from one plant generation to the next makes them exceptional candidates for biocontrol and growth enhancement applications [10,11,25]. Previous studies have illustrated that specific bacterial endophytes can directly stimulate the growth of host plants. They achieve this through nitrogen fixation, phosphorus solubilization, indole-3-acetic acid (IAA) synthesis, and siderophore production [15,22,26]. Additionally, these endophytic bacteria indirectly promote plant growth by inhibiting pathogenic activity. They produce antimicrobial agents, compete with pathogens for nutrients and habitats, and trigger the host plants to activate their defense mechanisms, thereby inducing systemic acquired resistance [23,24,27].

Native *Bacillus* isolates from tea rhizosphere soil for the control of tea anthracnose disease were identified and characterized. In vitro screenings using coculture assays revealed that eight *Bacillus*-like isolates significantly inhibited the mycelial growth of *C. fructicola*, with an inhibition exceeding 90%. This suggests their strong biocontrol efficacy against *C. fructicola*. Morphological, physiological, and biochemical assessments, along with molecular phylogenetic analysis, identified isolates T1 and T2 as *B. inaquosorum*; T3, T5, T7, T8, and T19 as *B. tequilensis*; and T6 as *B. spizizenii*. *Bacillus* species are known for their widespread presence and for the production of numerous antibiotic compounds effective against several plant pathogens, serving as alternatives to chemical fertilizers and synthetic pesticides [20,28]. *B. subtilis* strains, in particular, are notable for their broad-spectrum antibacterial and antifungal activity, stress resistance, resilient adaptability, and environmentally friendly properties, making them promising bio-active agents for disease control [29,30]. Recent studies have demonstrated that *B. tequilensis* and other *Bacillus* species can suppress the growth of *C. acutatum* and *C. capsici*, thereby protecting pepper plants from anthracnose disease through the production of antibiotic compounds [31,32]. Avery et al. (2020) demonstrated that *B. inaquosorum* strain T1 inhibited the growth of a *Vibrio* strain, causing an acute hepatopancreatic necrosis by inducing a gene cluster associated with antibacterial compound production [33]. Kamali et al. (2022) demonstrated, through integrated genome mining and chemical analysis, that *B. inaquosorum* KR2-7 protects against tomato *Fusarium* wilt [34]. Wu et al. (2024) reported that *B. altitudinis* GS-16 isolated from tea leaves exhibited antagonistic activity against tea anthracnose disease. In this study, *B. inaquosorum* strains (T1 and T2), *B. tequilensis* strains (T3, T5, T7, T8, and T19), and *B. spizizenii* strain T6 demonstrated the ability to secrete hydrolytic enzymes such as glucosidase, galactosidase, β-xylosidase, and amino acid hydrolase. These strains exhibited inhibitory activity against different strains of *C. fructicola*, achieving an average control level of 60.68% for tea anthracnose. This highlights the potential of these native endophytic bacteria as promising biocontrol agents against tea anthracnose.

*Bacillus* species are notable for producing secondary metabolites, enzymes, and antimicrobial proteins that disrupt the cellular structures of pathogens, inhibiting their growth and reducing their pathogenicity [10,23,25]. Peptide antibiotics, synthesized via non-ribosomal peptide synthetase pathways, exhibit strong antifungal activity, effectively controlling phytopathogens and reducing plant diseases, which indirectly promotes plant growth [22,35]. For instance, *B. velezensis* D produces antimicrobial substances like iturin, fengycin, surfactin, and enzymes that strongly inhibit *R. solanacearum* [36]. Similarly, *B. inaquosorum* KR2-7 has been shown to combat *Fusarium* wilt in tomato plants by producing nine key antifungal and antibacterial compounds, including fengycin, surfactin, bacillomycin F, bacillaene, and macrolactin, as identified through genome mining [34,35]. Additionally, *B. tequilensis* PKDN31 and *B. licheniformis* PKDL10 enhance tomato resistance against *Fusarium oxysporum* by boosting defense-related enzymes such as β -1,3 glucanase, polyphenol oxidase, and chitinase, as well as by increasing phenol content [37]. *B. subtilis* V26 significantly reduced potato dry rot by 54.8–60.8%, while promoting plant growth through the production of protease, glucanase, cellulase, and a variety of metabolites, including lipopeptides and polyketides [15]. These findings highlight that the antibacterial efficiency of *Bacillus* strains is closely linked to their production of essential biosynthetic gene clusters. In the current study, biocontrol marker genes responsible for the biosynthesis of polyketides and lipopeptides in *B. inaquosorum* (T1 and T2), *B. tequilensis* (T3, T5, T7, T8, and T19), and *B. spizizenii* (T6) isolates were identified through PCR analysis. This allows us to hypothesize that the biocontrol effect of these strains on *C. fructicola* is closely linked to the synthesis of antimicrobial substances like iturin, fengycin, subtilosin, and alkaline protease. In future studies, integrated omics approaches and mutagenesis of these strains will be employed to confirm the proposed modes of action and elucidate the exact functions of the putative genes and gene clusters involved in the suppression of the fungal pathogen *C. fructicola*.

Lastly, the presented results indicated that *B. inaquosorum*, *B. tequilensis*, and *B. spizizenii* isolates produce hydrolytic enzymes such as proteases, cellulases, and β-1,3-glucanases. These findings are consistent with previous studies on *Bacillus* spp., confirming their significant potential as biocontrol agents against tea anthracnose disease.

## 4. Materials and Methods

### 4.1. Fungal and Bacterial Strains

From June to September 2020–2023, tea anthracnose disease was investigated at key tea cultivation sites in Fujian to assess the progression of the disease. In each tea garden, the tea bushes exhibited the typical dieback symptoms associated with anthracnose. During the initial stages of infection, the base of the stems and leaves of the plucking shoots presented circular to elongated dark brown lesions (Figure 1A). As the disease progressed, these lesions expanded upward, leading to significant necrosis of the plant tissues (Figure 1B). Ultimately, the disease hampers shoot growth, and white perithecia develop on the lesions, which can persist in the soil and crop debris for several years. Chen et al. isolated the pathogenic fungus from diseased tea leaves and verified them as *C. fructicola* (Figure 1C-1D) [22], preserving the samples at the Fujian Provincial Key Laboratory for Control of Forest Disease and Pests, located at Ningde Normal University (Ningde, China). Bacterial stains were isolated from soil samples taken from tea plantation in Zherong (27°25′35.7″ N, 119°8′36.4″ E) and Huotong (26°8′9.11″ N, 119°3′8.61″ E), both located in Ningde, China. Samples were collected between 5 cm and 20 cm above the surface, with three replicates at each site.

### 4.2. Isolation and Assessment of Antagonistic Bacterial Strains against C. fructicola

The soil samples were collected from the rhizosphere of tea plantations affected by anthracnose disease. To isolate *Bacillus* strains, 5.0 g of rhizosphere soil was homogenized in 45 mL of dilution water by shaking for 30 min. Subsequently, 200 uL of the homogenized material, which was diluted to 10^−2^, 10^−3^, and 10^−4^, was spread on potato dextrose agar (PDA) plates and then incubated at 37 °C for 48 h (Figure 1E), with the last rinse water used as a control. Based on colony characteristics, a total of 38 *Bacillus*-like colonies were isolated on the LB plates using the streaking inoculation method, and the colonies were stored at −20 °C.

A 5 mm diameter mycelial disc from the growing edge of 7-day-old colonies of *C. fructicola* was selected and positioned at the center of the PDA plates. Each bacterial isolate was then inoculated at four equidistant points 20 mm away from the pathogen, with the pathogen disc alone acting as a control. This procedure was repeated three times for each plate. Following an incubation period at 37 °C for 7 days, the diameters of the *C. fructicola* colonies was measured, and the inhibition rates were determined using the following formula: inhibition rate = (C − T)/(C − 5) × 100%, with C and T representing the respective colony diameters of the pathogen in the absence or presence of the tested bacteria.

### 4.3. Morphological, Physiological, and Biochemical Assessments of Bacterial Isolates

The *Bacillus*-like isolates (T1, T2, T3, T4, T5, T7, T8, and T19) were subjected to an initial analysis of phenotypic properties, which included examining colony morphology, Gram reaction, endospore formation, and physiological and biochemical characteristics [22,30]. Furthermore, these isolates underwent identification through sequencing of the housekeeping gene fragments of 16S rRNA. The partial gene sequences were amplified by PCR (30 cycles) and sequenced using the primers listed in Supplementary Table S1. The sequencing results were aligned using DNAMAN software and compared against nucleotide sequences in the GenBank database utilizing BLAST. Subsequently, a phylogenetic tree was constructed using the neighbor-joining method with MEGA 11.0 software.

Eight isolates were selected for analysis using the VITEK^®^ 2 systems, in conjunction with conventional methods [30]. The investigation of carbon source utilization was conducted through biochemical tests using test tubes. The physiological and biochemical tests primarily involved the white line in agar test, which was carried out in triplicate for each assessment.

### 4.4. DNA Extraction and 16S rRNA Gene Amplification of Bacterial Isolates

Genomic DNA was extracted from bacterial isolates using the Tianamp Bacterial DNA Kit (Tiangen, Beijing, China). The 16S rRNA gene was amplified utilizing the primers listed in Table S1. The reaction mixture consisted of 10 μL of 2 × Pro Taq Master Mix (dye plus, Vazyme, Nanjing, China), 1 μg genomic DNA, 1 μL of each primer (10 μM), and 7 μL ddH_2_O. The PCR amplification program included an initial denaturation at 94 °C for 5 min, followed by 30 cycles of denaturation at 94 °C for 30 s, annealing at 55 °C for 30 s, extension at 72 °C for 1 min, and a final extension at 72 °C for 5 min. The PCR products were purified and sent to Qingke Biological Company (Fuzhou, China) for sequencing, using the primers listed in Supplementary Table S1. The generated sequences were submitted to NCBI for the assigning of accession numbers, and they also underwent homology alignment analysis using BLAST. A phylogenetic tree was constructed to classify their phylogenetic relationships using the maximum likelihood method in MEGA 11, based on the 16S rRNA gene sequences of the strains.

### 4.5. Antifungal Spectrum of Bacillus-like Isolates In Vitro

The tea anthracnose pathogen, namely *C. fructicola*, was employed to assess the antifungal efficacy of *Bacillus* isolates in vitro. The experiments were replicated, biologically and technically, in triplicate. The evaluation of the pathogen colony diameter and the inhibition rate was carried out following the procedure outlined in Section 4.2.

### 4.6. Molecular Identification of Genes Encoding Antimicrobial Compounds

Following DNA extraction, partial segments of genes encoding antimicrobial compounds in the strains, including alkaline protease (*aprE*), iturin (*ituA* and *ituC*), fengycin (*fenA* and *fenD*), and subtilosin-A (*sboA*), were amplified using the primers and annealing temperatures specified in Table S1. DNA from *Bacillus subtilis* served as the positive control, while ddH_2_O was used as the negative control.

The PCR reaction mixture consisted of 10 μL of 2 × Pro Taq Master Mix (dye plus, Vazyme, Nanjing, China), 1 μg of genomic DNA, 1 μL of each primer (10 μM), and 7 μL of ddH_2_O. The PCR amplification program was as follows: an initial pre-denaturation at 94 °C for 5 min, followed by 30 cycles of denaturation at 94 °C for 30 s, annealing at 55–61 °C (depending on the corresponding primers, listed in Table S1) for 30 s, and extension at 72 °C for 1 min, with a final extension at 72 °C for 5 min. The PCR products were analyzed by 1% agarose gel electrophoresis, with bands stained using ethidium bromide (EB) and visualized via a gel imaging system to confirm their sizes.

### 4.7. In Vivo Efficacy against Tea Anthracnose under Greenhouse Conditions

The plant protection assay was conducted in the greenhouse at Ningde Normal University, Ningde, China. Two-month-old healthy tea seedlings were grown in pots containing sterilized soil. The leaves were challenge-inoculated with agar discs (2.5 mm in diameter) containing the actively growing mycelium of *C. fructicola*. On the third day post-pathogen infection, the leaves were inoculated with 100 mL of the *Bacillus* spp. strains (10^7^ conidia/mL). A control group of leaves was inoculated solely with the pathogen for comparison. Each treatment included 15 leaves, and the experiment was conducted in triplicate. The seedlings were regularly watered and fertilized, maintained at 28 ± 2 °C with 85% relative humidity over a 10-day period. Symptoms of anthracnose wilt were monitored through visual observation and analyzed using a disease scoring system [6]. Disease incidence, including disease lesion area and biocontrol efficacy, was calculated to evaluate the relative severity of the disease. The lesion area was measured using Image J version 1.54j software, and biocontrol efficacy was calculated using the following formula: [(lesion area of control—lesion area of treatment)/lesion area of control] × 100.

### 4.8. Statistical Analyses

The experimental data underwent analysis using a one-way analysis of variance (ANOVA) using SPSS 24.0. All data are presented as the mean ± standard error. Significant differences (*p* < 0.05) in mean values between the control group and isolate treatments were further examined using a Duncan’s multiple range test.

## 5. Conclusions

This study provides significant insights into managing *Colletotrichum fructicola*-induced diseases in tea plants. We assessed bacterial isolates from the tea rhizosphere that had previously been identified for their antagonistic activity against various pathogens. Of the eight isolates tested, *Bacillus tequilensis* T3 was the most effective, demonstrating strong efficacy in both in vitro and greenhouse experiments. Moreover, these isolates activated the expression of genes associated with antimicrobial compounds, effectively inhibiting the mycelial growth of *C. fructicola*, with *B. tequilensis* T3 showing the highest level of inhibition. Additional large-scale tests are necessary to further investigate and clarify the exact roles of the implicated genes and gene clusters in the suppression of *C. fructicola* by these promising biocontrol agents. Overall, this research offers valuable perspectives on tea anthracnose management strategies, highlighting the potential of *B. inaquosorum*, *B. tequilensis*, and *B. spizizenii* strains as effective biocontrol agents. 

## Figures and Tables

**Table 1:** Inhibitory effects of *Bacillus*-like isolates against tea anthracnose pathogen *C. fructicola*.

Treatment	Colony Diameter of Isolates (cm)	Diameter of the Zone (cm)
CK	6.37 ± 0.68	6.25 ± 0.66
T1	5.87 ± 0.71	3.57 ± 0.32
T2	6.19 ± 0.82	3.55 ± 0.49
T3	5.71 ± 1.91	3.33 ± 0.15
T5	6.25 ± 0.53	3.79 ± 0.42
T6	5.99 ± 0.55	3.81 ± 0.14
T7	5.48 ± 0.78	3.79 ± 0.24
T8	5.72 ± 0.51	3.36 ± 0.12
T19	5.83 ± 0.66	3.60 ± 0.42

**Table 2:** Physiological and biochemical characteristics of eight *Bacillus* isolates.

Characteristic	Isolates							
	T1	T2	T3	T5	T6	T7	T8	T19
Hydrolytic enzymes								
Glucosidase	+	+	+	+	+	+	+	−
Mannosidase	−	−	−	−	−	−	−	−
Galactosidase	+	+	+	+	+	+	+	−
β−Xylosidase	+	+	+	+	+	+	+	−
β−N−Acetyl−Glucosaminidase	−	−	−	−	−	−	−	−
Alanine Arylamidase	+	+	−	−	−	+	−	−
Aspartate Arylamidase	−	−	+	−	−	−	−	−
Ala−Phe−Pro Arylamidase	−	−	−	+	+	+	−	+
Glycine arylaminase	+	+	+	+	−	−	+	−
Lysine Arylamidase	−	−	−	−	−	−	−	−
Leucine Arylamidase	+	+	+	+	+	+	+	−
Phenylalanine Arylamidase	+	+	+	+	+	+	+	−
Proline Arylamidase	−	−	−	−	−	−	−	−
Pyrrolidonyl Arylamidase	+	+	+	+	+	+	+	+
Tyrosine Arylamidase	+	+	+	+	+	+	+	−
Esculin Hydrolyse	+	+	+	+	+	+	+	−
Biochemical compounds								
D−Glucose	+	+	+	+	+	+	+	−
D−Galactose	−	−	−	−	−	+	−	−
D−Ribose	−	−	−	+	+	+	+	−
Maltotriose	+	−	−	+	+	+	+	−
D−Mannose	+	+	+	+	+	+	+	−
D−Melezitose	−	−	−	−	−	−	−	−
D−Tagatose	−	−	−	−	−	+	−	−
D−Trehalose Dihydrate	+	+	+	+	+	+	−	−
Glycogen	−	−	−	−	−	+	−	−
L−Rhamnose	−	−	−	−	−	+	−	−
Cyclodextrin	+	−	+	−	−	−	−	−
D−Mannitol	+	+	+	+	+	+	+	−
Inositol	+	+	+	+	+	+	+	−
D−Methylglucoside	+	+	+	+	+	+	−	−
Methyl−D−Xylopyranoside	−	−	−	−	−	−	−	−
N−Acetyl−D−Glucosamine	−	−	−	−	−	−	−	−
Inulin	+	+	+	+	+	+	+	−
Phosphoryl Vitamin C	−	−	−	−	−	−	−	−
Pyruvate	+	+	+	+	+	+	+	−
Resistance traits								
Putrescine (PUT) Assay	−	−	−	−	−	−	−	−
NaCl 6.5%	+	+	+	+	+	+	+	−
Kanamycin	+	+	+	−	−	−	−	−
Oleandomycin Phosphate	−	+	−	−	−	+	−	−
Polymyxin B	+	+	+	+	+	+	+	−

Note: + and − represents positive and negative results, respectively.

**Table 3:** Inhibition rates of different *Bacillus* isolates on growth of diverse strains of *C. fructicola*.

Pathogens	Inhibition Rate of *Bacillus* Isolates (%)							
	T1	T2	T3	T5	T6	T7	T8	T19
*C. fructicola* strain N1#	65.49	60.71	97.36	45.09	44.08	54.41	59.45	68.26
*C. fructicola* strain N5#	56.43	66.64	63.32	59.17	64.56	68.46	67.80	60.49
*C. fructicola* strain N8#	67.03	51.63	48.89	59.28	57.20	52.01	60.60	57.86

## Data Availability

The data that support the findings of this study are available from the corresponding author upon reasonable request.

## Supplementary Materials

The following supporting information can be downloaded at: https://www.mdpi.com/article/10.3390/plants13202889/s1, Figure S1: The PCR products of the 16S rRNA gene using genomic DNA of eight Bacillus isolates were verified using a 1% agarose gel. M, size marker; 1–8, 16S rRNA bands of T1, T2, T3, T5, T6, T8, and T19, respectively; Table S1: Primers used in this study.
