# Supplementary material for: Evaluating Native Bacillus Strains as Potential Biocontrol Agents against Tea Anthracnose Caused by Colletotrichum fructicola
Source: Plants (Basel). 2024 Oct 15;13(20):2889. doi: 10.3390/plants13202889 (PMC11511046; doi:10.3390/plants13202889)

Supplemental materials

Supplemental Table 1. Primers used in this study.

Gene name	Primer sequence (F/R)	Annealing temperature
16S rRNA	AGAGTTTGATCCTGGCTCAG<br>GGGTGAGCTGCAAACTTCTC	55 ℃
sboA	TCGGTTTGTAAACTTCAACTGC<br>GGGAGATCCTTACCCCCATA	58 ℃
aprE	AACTCAATCGGTGTTCTGGG<br>ATTGTGCAGCTGCTTGTACG	58 ℃
fenA	AAGCAGGCAGGAGCAAATTA<br>CTCAGTTGAGCGTTCCATCA	55 ℃
fenD	GACGAGCTTGTTTACTCCGC<br>GCTTGCCGAAGAACAGTTTC	55 ℃
ituA	TGCCAGACAGTATGAGGCAG<br>CATGCCGTATCCACTGTGAC	61 ℃
ituC	CCGTAATCAACCGTCTCGTT<br>GGGTGAGCTGCAAACTTCTC	58 ℃

Supplemental Figure 1. The PCR products of 16S rRNA gene using genomic DNA of eight *Bacillus* isolates were verified by a 1% agarose gel. M, Size marker; 1–8, 16S rDNA bands of T1, T2, T3, T5, T6, T8 and T19, respectively.

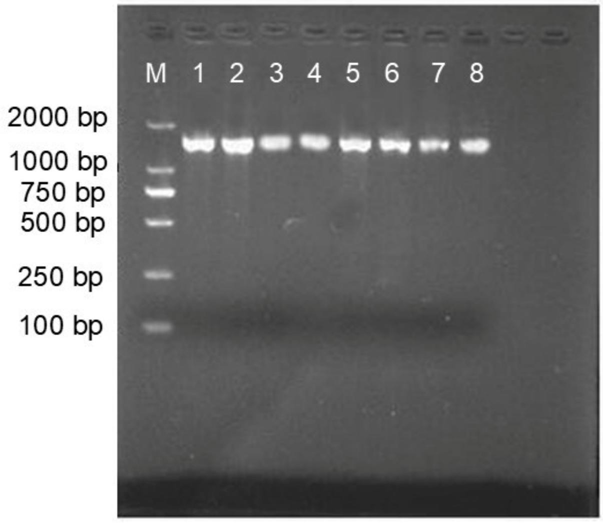

Supplement: Supplementary file 1 [file plants-13-02889-s001.zip › plants-3222584-supplementary.pdf]
